# Nurse-led video-coaching interventions in childhood, adolescent and young adult cancer survivors (REVIVER): a protocol for mixed methods feasibility research

**DOI:** 10.1186/s40814-019-0535-1

**Published:** 2019-12-18

**Authors:** Eline Bouwman, Rosella P. M. G. Hermens, Nicole M. A. Blijlevens, Judith B. Prins, Jacqueline J. Loonen

**Affiliations:** 1 Centre of Expertise for Cancer Survivorship, Department of Haematology, Radboud University Medical Centre, Geert Grooteplein Zuid 10 (Route 558), 6525 GA Nijmegen, NL Netherlands; 2 Radboud Institute for Health Sciences (RIHS), Scientific Institute for Quality of Healthcare (IQ Healthcare), Radboud University Medical Centre, Geert Grooteplein Noord 21 (Route 114), 6525 EZ Nijmegen, Netherlands; 3 Department of Haematology, Radboud University Medical Centre, Geert Grooteplein Zuid 8 (Route 476), 6525 GA Nijmegen, Netherlands; 4 Department of Medical Psychology, Radboud University Medical Centre, Geert Grooteplein Zuid 10 (Route 840), 6525 GA Nijmegen, Netherlands

**Keywords:** Childhood, Adolescent and young adult cancer survivors, eHealth, Cancer-related fatigue, Self-efficacy, Self-management, Lifestyle, Person-centred care, Cognitive behaviour therapy, Motivational interviewing, Nurse-led interventions

## Abstract

**Background:**

Successful cancer treatment can lead to cancer survivors being predisposed to an increased lifelong risk of adverse late health effects. Therefore, high-quality cancer survivorship care to earlier detect and treat late effects or to preserve survivor’s health is essential. Nevertheless, this care needs to be sustainable and cost-effective as well. We developed three different screen-to-screen nurse-led eHealth interventions for survivors of childhood, adolescent and young adult-onset cancer, collectively called the REVIVER interventions. Elaborating on person-centred care principles with content based on cognitive behavioural therapy modules and/or motivational interviewing techniques, these interventions aim to empower and coach survivors to improve (1) symptoms of cancer-related fatigue, (2) self-efficacy and self-management or (3) lifestyle. With the REVIVER study, we aim to evaluate the interventions’ feasibility and gain insights into the potential effectiveness.

**Methods:**

The REVIVER study involves a mixed methods design, including (1) interviews till data saturation with cancer survivors who completed the interventions as well as with all involved medical professionals, (2) reviews of nurses reports and (3) a single-group, pre-post evaluation among cancer survivors. Eligible survivors are survivors of childhood, adolescent and young adult-onset cancer who are referred to one of the interventions, in complete remission of cancer, 16–44 years old at enrolment, completed treatment at least 5 years ago and have access to a device with Internet options. We will assess feasibility in terms of demand, adherence, acceptability, practicality and integration/implementation. Health-related quality of life, as primary outcome of the potential effectiveness evaluation, will be assessed at three different time points: prior to the intervention; immediately following the intervention and 6 months post-intervention. Secondary outcome measures include changes in level of fatigue, self-efficacy, self-management and lifestyle.

**Discussion:**

This is the first study to evaluate the feasibility and potential effectiveness of eHealth nurse-led interventions elaborating on person-centred care, using cognitive behavioural therapy and/or motivational interviewing techniques as an innovative and promising approach for providing CAYA cancer survivorship care. If the interventions prove to be feasible and potential effective, a randomized controlled trial will be conducted to test the (cost)-effectiveness.

## Background

In the last decades, worldwide survival rates for patients with cancer have improved. As a result, the number of cancer survivors is rapidly increasing [1–3]. However, a disadvantage of successful treatment is that, due to chemotherapy and/or radiotherapy, cancer survivors are predisposed to an elevated lifelong risk of late adverse health effects [4–7]. These late effects, which may appear even years or decades after treatment, can be serious, leading to chronic morbidity and premature mortality [5].

### Late effects of cancer treatment

Late effects of cancer treatment can have a negative impact on multiple dimensions of health, including physical and psychosocial health. Cancer-related fatigue (CRF), one of the most common treatment-related late effects, is known to seriously hamper survivors’ daily life activities such as attending school or work [8]. Another category of common late effects involves cardiovascular diseases caused by anthracyclines and/or radiotherapy over the chest [9–13]. Moreover, survivors treated with cranial irradiation are at an increased risk of developing endocrine disorders related to obesity [14]. Furthermore, it is now well established from a variety of studies that also health-related quality of life (HRQOL) of survivors can be severely compromised by the development of these and other late effects resulting from their past treatment [15–18]. The manifestation of these health conditions in cancer survivors can be negatively influenced by the presence of unfavourable lifestyle risk factors such as overweight, smoking behaviours and/or a low physical activity level. These concerns stress the need to adopt or to continue a healthy lifestyle for this population. This is supported by Jones et al., who found that in adult survivors of childhood-onset Hodgkin lymphoma, exercise can lower their risk of cardiovascular events in a dose-dependent manner [19].

### Cancer survivorship care

The nature and incidence of late effects underscore the need for high-quality long-term follow-up care for cancer survivors. Therefore, the Centre of Expertise for Cancer Survivorship in the Netherlands developed the innovative Personalized Cancer Survivorship Care model with three important purposes: (1) to earlier detect and (2) treat late effects or (3) to preserve survivor’s health [20]. Care within this model is delivered according to the principles of person-centred care (PCC) as developed by Ekman et al. [21]. Partnership between the patient and the medical professional is the most important feature in PCC. This partnership, based on an equal footing, gives the patient an active role in his or her own care. Hence, PCC is thought to be an important contributing factor in promoting self-efficacy and self-management. Stimulating self-efficacy and self-management helps patients to take control of their own lives and to obtain a higher quality of life [22]. This illustrates the necessity of incorporating PCC in cancer survivorship care.

### eHealth interventions

Given limited healthcare resources, provision of follow-up care needs to be both sustainable and cost-effective [23]. Electronic health (eHealth) interventions are accessible to cancer survivors from a home situation, making eHealth an attractive means to deliver interventions with limited resources [24]. In addition to the cost-effectiveness promises of eHealth interventions, they can also be favourable for survivors as they are released from unnecessary clinic visits that may hinder the survivors’ daily life activities [25]. Post and Flanagan report in their integrative review potential for web-based survivorship interventions to be feasible and acceptable in breast cancer survivors [25]. In addition, a study of Abrahams et al. found Internet-based cognitive behavioural therapy to be accessible and effective in reducing severe fatigue and related symptoms in breast cancer survivors as well [26].

### REVIVER interventions

In order to treat survivors for late effects of cancer treatment in a cost-effective way with limited burden, an eHealth solution for survivors of childhood, adolescent and young adult (CAYA) cancer was developed and has recently been implemented at the Centre of Expertise for Cancer Survivorship. These so-called REVIVER interventions, with PCC as core principle, are part of cancer survivorship care to improve the following direct or indirect late effects of cancer treatment: CRF, self-efficacy and self-management or lifestyle. The interventions are led by a trained nurse who applies either cognitive behaviour therapy (CBT), motivational interviewing (MI) or a combination of both to help survivors overcome their late effects. However, there is a need for feasibility and potential effectiveness evaluations in order to implement the REVIVER interventions on a wider scale.

Our primary study objective relates to the assessment of the feasibility of the REVIVER interventions and can be broken down into two specific objectives:

1. Determine feasibility of the REVIVER interventions for CAYA cancer survivors, in terms of:

- Demand
- Adherence to the sessions
- Acceptability (e.g. content and delivery)
- Practicality (e.g. mode of delivery)
- Integration/implementation (e.g. facilitators and barriers)

2. Determine feasibility of the REVIVER interventions for the medical professionals, in terms of:

- Acceptability (e.g. content, delivery and referral to interventions)
- Practicality (e.g. mode of delivery)
- Integration/implementation (e.g. facilitators and barriers)

Our secondary objective is to gain insight into the potential effectiveness of the REVIVER interventions in CAYA cancer survivors, in terms of quality of life, fatigue, self-efficacy, self-management and lifestyle.

## Methods

The protocol of the REVIVER study is drafted according to the COREQ checklist and STROBE Statement [27, 28].

### Design

**Fig. 1:**
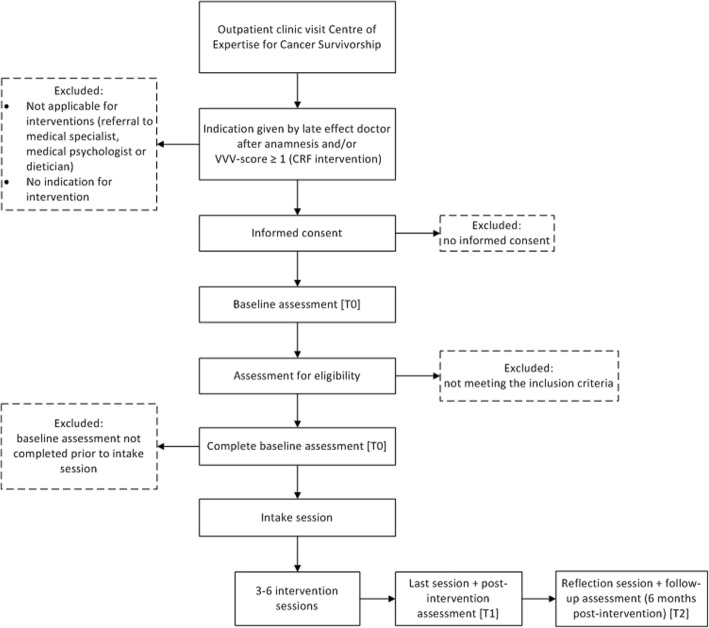
Flowchart REVIVER study

The REVIVER study will involve a mixed methods research approach. Feasibility will primarily be assessed with qualitative measures, i.e. semi-structured interviews. Quantitative measures will be used to determine adherence to and gain insight into the potential effectiveness of the REVIVER interventions. Therefore, we will apply a single arm pre- and post-test design, with three different measurement points: baseline (T0), following the last session (T1) and 6 months post-intervention (T2). Figure 1 outlines the stages of participation in the REVIVER study interventions.

### Study population

#### CAYA cancer survivors

Our primary study population involves CAYA cancer survivors who are at least 5 years post diagnosis. They are eligible for the REVIVER study if they (i) are referred to one of the REVIVER interventions (see Table 1); (ii) are in complete remission of cancer; (iii) are 16–44 years old at enrolment; (iv) are having a basic proficiency in the Dutch language; (v) are having access to a device with Internet options (i.e. smartphone or tablet); and (vi) have given informed consent. We will exclude cancer survivors (i) whose symptoms of fatigue may be caused by an underlying medical condition as these patients need different treatment (CRF intervention); (ii) suffering from complex endocrine disorders explaining overweight (lifestyle intervention); (iii) suffering from serious cognitive or psychological problems; and/or (iv) participating in an intervention study or other interventions aiming at improving CRF, low self-efficacy, self-management or lifestyle.

**Table 1:** Criteria for referral to the nurse-led video-coaching interventions (REVIVER)

For referral to the nurse-led video-coaching interventions, participants must:	
• Be a survivor of childhood, adolescent or young-adult cancer (diagnosed with any type of cancer under the age of 39)	
• Completed treatment with chemotherapy and/or radiation therapy for CAYA cancer (with or without surgery, with or without haematopoietic stem cell transplantation) and/ or treatment for a brain tumour at least 5 years ago	
• Visited the Centre of Expertise of Cancer survivorship outpatient clinic at least once	
• Received one of the following indications:	
o Indication of moderate to severe symptoms of cancer-related fatigue. This can be defined in two ways:	
1. Fatigue score ≥ 18 assessed by the Short Fatigue Questionnaire [29]	
2. Fatigue interfering with daily life activities and fatigue lasting at least 6 months	
o Indication of need for more empowerment. This can be defined in two ways:	
1. General Self-Efficacy Scale score ≤ 29 [30]	
2. Late effect doctor of the Centre of Expertise for Cancer Survivorship indicates a low empowerment state of the survivor after anamnesis during consultation at the outpatient clinic	
o Indication of present unhealthy lifestyle factors given by late effect doctor of the Centre of Expertise for Cancer Survivorship outpatient clinic after anamnesis. Unhealthy lifestyle factors include:	
1. A BMI of ≥ 25	
2. Smoking	
3. A low physical activity level	

#### Medical professionals

The second study population is consisting of medical professionals affiliated with the Centre of Expertise for Cancer Survivorship Care and involved with the REVIVER interventions. This sample includes nurses and doctors with late effect expertise and members of the psychosocial expert team.

### Sample size

#### Feasibility evaluation

For feasibility evaluation with semi-structured qualitative interviews, we will recruit a small group of CAYA survivors from the survivors participating in the REVIVER study for potential effectiveness evaluation by means of purposive sampling. The exact number of participating CAYA survivors is dependent on data saturation, which is expected to occur after interviewing approximately 15 CAYA survivors. In addition, all medical professionals (N = 9) involved in CAYA survivorship care at the Centre of Expertise for Cancer Survivorship Care will be recruited for qualitative interviews, including late effect nurses (N = 2) and doctors (N = 4), respectively, and members of the psychosocial expert team (N = 3).

#### Potential effectiveness evaluation

The REVIVER study was primarily designed for feasibility evaluation of the REVIVER interventions. Our secondary aim is to gain insight into the potential effectiveness of the interventions. We estimate that a total of 60 CAYA cancer survivors with 20 survivors per type of intervention is feasible to reach this aim.

This sample size is realistic considering the number of CAYA survivors invited to the outpatient clinic at the Centre of Expertise for Cancer Survivorship per month (± 48) and the study running time of 24 months. Approximately 1 out of 10 CAYA survivors will be eligible for the interventions and be referred to the REVIVER interventions, resulting in a total of 116 eligible survivors after 24 months. Assuming a recruitment rate of 50%, a sample size of 60 will allow us to gain sufficient insight into potential effectiveness of the REVIVER interventions.

### REVIVER interventions

The REVIVER interventions are designed for CAYA survivors to receive coaching to cope with direct or indirect late effects of cancer and are delivered by qualified nurses via secured screen-to-screen video calling software. The interventions, elaborating on PCC principles, are aimed at improvement of (i) symptoms of CRF, (ii) self-efficacy and self-management or (iii) lifestyle. The REVIVER interventions consist of an intake, 3 to 6 screen-to-screen video-coaching sessions and a reflection session. On average, the intake session as well as the coaching sessions will be delivered within a 3-month time period. After a 6-month period in which the survivor can actively work on his or her goals set during the coaching sessions, a reflection session will follow. Depending on the type of intervention, the content is based on CBT and/or MI techniques (Table 2). Fidelity of the REVIVER interventions will be pursued in the three following ways:

**Table 2:** REVIVER interventions

Goal	To empower, to motivate and to coach CAYA survivors to actively work on improving and managing either CRF, self-efficacy and self-management or lifestyle
Type	Individual eHealth nurse-led video-coaching interventions delivered during screen-to-screen sessions
Duration	An intake session, 3–6 coaching sessions over a time course of approximately 3 months, and a 6-month follow-up reflection session. Average duration of each session is 30–45 min
Basic principle	Person-centred care
Structure	
• Phase 1 (intake) *Engaging with survivor/focus setting*	During the intake, the nurse will reflectively listen to the survivor’s narrative, try to build a mutually trust with the survivor and explore the survivor’s stage of change. Focus for the following sessions will be discussed
• Phase 2 (3–6 sessions) *Coaching with evidence-based modules of CBT and/or MI*	According to needs and preferences of the survivor, 3–6 coaching sessions will follow Content of the interventions: o *CRF*: module of cognitive behaviour therapy light for chronic fatigue after cancer [31] o *Empowerment*: module cognitive behaviour therapy on four-phase recovery for cancer survivors and motivational interviewing for goal setting [32] o *Lifestyle*: motivational interviewing according to the stage of change of survivor [33]
o Phase 3 (reflection session) *Reflection on last period, sustainable goal setting*	During the reflection session, the survivor’s progress will be reviewed, and if needed, new strategies will be made to still achieve the goals made earlier. In addition, plans will be made to make it a sustainable goal

#### 1.Competence of nurses

The nurses are qualified to deliver the REVIVER interventions based on CBT and/or MI techniques by following a training on CBT and a certified course on MI techniques. In addition, to evaluate the CBT coaching sessions, the nurses have regular (approximately once every 2 months) peer-to-peer coaching with a medical psychologist with experience on CBT.

#### 2.Self-reported adherence of nurses to intervention protocol

Following every intake, coaching or reflection session, the nurses are instructed to fill out an online checklist to check for adherence to the intervention protocol. The checklist includes questions on, for example, the survivor’s story (i.e. stage of change) and facilitators and barriers perceived by the nurses.

#### 3. Adherence of nurses to intervention protocol according to survivors

Following the last coaching sessions, a number of survivors are asked to participate in an interview on facilitators and barriers of the interventions. In this interview, survivors are also asked on the important components of the intervention protocol to check adherence of the nurses to the protocol.

### Study procedures

CAYA cancer survivors will be mainly recruited by late effect doctors affiliated with the Centre of Expertise for Cancer Survivorship while attending a regular medical follow-up consultation at the outpatient clinic. During consultation, when applicable, the REVIVER interventions will be introduced and discussed by the doctor.

Another flow of survivors is coming from the psychosocial expert team of the Centre of Expertise for Cancer Survivorship Care. The medical psychologists or occupational physician may decide, after treatment and/or consultations, to refer the CAYA survivor to the interventions as well. The decision to refer the survivor to the interventions will always be based on shared decision-making. After referral to the interventions, the nurses of the REVIVER interventions will inform the survivor on the REVIVER study and ask consent to participate in the study.

All questionnaire assessments for each measurement moment (T0, T1 and T2) and intervention type are listed in Table 3.

**Table 3:** Schedule with specific measurements for survivors for each measurement moment

	Average min to complete	REVIVER interventions		
		T0	T1	T2
*Questionnaires*				
• Standard anamnesis questionnaire	20	X		
• Health-related quality of life (QLQ-C30) [34]	5–10	X	X	X
• Fatigue (CIS20r) [35]	5–10	X	X	X
• Self-efficacy (General Self-Efficacy Scale) [30]	5–10	X	X	X
• Self-management (SeMaS scale) [36]	5–10	X	X	X
• Lifestyle (Leefstijlvragenlijst) [37–40]	15–20	X	X	X
• Physical activity (SQUASH questionnaire) [41]	3–5	X	X	X
*Interviews*				
• Interview experiences REVIVER interventions^*^	45–60		X	

^*^Only applicable to a small sample (N = 10–15) of survivors participating in the evaluation part of the REVIVER study

#### Feasibility evaluation

The survivors for feasibility evaluation will be recruited from the sample of survivors of the potential effectiveness evaluation part of the REVIVER study. During the last video calling session of the REVIVER intervention, the nurse will approach survivors to ask consent to participate in an interview and to be audio recorded. The interviews will take place in the weeks following the last session of the intervention. Beforehand, the researcher will explain the goals for doing this research. Average duration of each interview will be approximately 45–60 min.

Likewise, at the end of the study period, interviews with an average duration of 30–45 min will be conducted with the medical professionals by the same researcher with the medical professional’s consent for audio to be recorded.

An interview guide for the survivors will be developed by the researchers to (semi)structure the interviews (available on request). The guide will contain questions on mode of delivery, content and delivery and facilitators and barriers of the interventions. It will conclude with the survivors’ intentions to continue using the learned strategies to cope with symptoms of fatigue and low self-efficacy level or to adopt/maintain a healthy lifestyle in daily life. For medical professionals, the focus of the guide will be on the practicality of the interventions, fit within the organizational culture and perceived facilitators and barriers. The interview guide will be tested as a pilot in CAYA cancer survivors and other medical professionals affiliated with the Centre of Expertise for Cancer Survivorship. The survivors and medical professionals will be interviewed by an independent researcher who is not involved with the interventions. Both the survivors and medical professionals will be provided with a summary of the results following the end of the study. The nurses’ reports, written after each session with the survivor, will be analysed to assess adherence to the interventions.

These reports contain information on duration and frequency of the sessions, type of module/phase of MI, goals set and goals completed by the survivor. In addition, data on perceived facilitators and barriers of the REVIVER intervention will be collected from the reports as well.

#### Potential effectiveness evaluation

To gain insight into the potential effectiveness of the REVIVER interventions, data from different sources will be extracted. First, we will collect socio-demographic and clinical information from the medical records. Secondly, to assess information on the amount and duration of the sessions needed per survivor, data from the nurses’ reports will be extracted.

Lastly, the survivors are provided with questionnaires at three different time points; prior to the sessions at baseline (T0), immediately following the last session (T1) and at 6 months follow-up (T2). As part of care as usual at the Centre of Expertise for Cancer Survivorship, the General Self-Efficacy (GSE) Scale is incorporated in the standard anamnesis questionnaire that is provided to every survivor attending the outpatient clinic. Therefore, a baseline measurement of self-efficacy can be derived from the anamnesis questionnaire which is incorporated in the survivor’s medical record.

### Outcome measures

#### Feasibility evaluation

The outcome measures for both samples of participants will be mainly based on guidelines from Bowen et al. to assess the feasibility of the REVIVER interventions, including [42]:

- *Demand*
  Outcomes of interest for demand of the interventions include whether the survivors prefer the REVIVER intervention to care as usual, whether the survivors intend to continue applying the content of the interventions in daily life and whether the medical professionals perceive demand for using the REVIVER interventions in daily practice.
- *Adherence of survivors with interventions (actual use)*
  Outcome of interest for adherence with the interventions includes the percentage of planned sessions joined by the survivors.
- *Acceptability*
  Outcomes of interest include the satisfaction and perceived appropriateness in both survivors and medical professionals with the content and delivery of the REVIVER interventions and fit within the organizational culture.
- *Practicality*
  Outcomes of interest include the experiences with the mode of delivery of the REVIVER interventions for both survivors and medical professionals in terms of efficiency, ability to carry out the intervention activities and positive and negative effects on the survivors
- *Integration/implementation of the REVIVER interventions*
  Outcomes of interest include success or failure of the REVIVER interventions and perceived facilitators and barriers of success or failure of the REVIVER interventions for both survivors and medical professionals.

#### Potential effectiveness evaluation

##### Health-related quality of life

To evaluate the potential effectiveness of the interventions, HRQOL will be assessed in survivors through the disease-specific EORTC quality of life questionnaire (QLQ-C30) [34]. It includes five domains: a functional scale (physical, role, emotional, cognitive, social), and several symptom scales (fatigue, nausea and vomiting, pain, dyspnoea, insomnia, appetite loss, constipation, diarrhoea, financial difficulties) are incorporated in the QLQ-C30 questionnaire. It concludes with 2 items to assess the survivor’s global health status. All items, with the exception of the global health status items, are scored on a 4-point Likert-type scale with the response alternatives “not at all”, “a little”, “quite a bit” and “very much”. Global health status is scored on a 7-point scale at which the patients can indicate how they perceive their own health status and quality of life with 1 equalling “very poor” to 7 equalling “excellent”. Higher scores on the functional scale and global health status indicate a higher HRQOL. Internal consistency of the QLQ-C30 has shown to be high with a Cronbach alpha of 0.95 and 0.94 for healthy people and patients with cancer, respectively [43, 44].

##### Fatigue

Level of fatigue will be determined in survivors with the generic CIS20R questionnaire [35]. It assesses fatigue with four dimensions, including subjective experience of fatigue and reduction in motivation, activity and concentration. A 7-point Likert scale is used to score the items. Survivors are asked to rate how strongly they agree with each statement. The CIS20R has been shown to be a valid and reliable tool, with Cronbach alpha ranging from 0.84 to 0.95 in the Dutch general population and groups with diverse medical conditions, including cancer survivors [45].

##### Self-efficacy

The GSE Scale gives a rough estimation of the survivor’s self-efficacy level [30]. Survivors will be asked to indicate to what extent the 10 items apply to how they think or act in certain situations. All questionnaire items utilize a 4-point Likert-type scale, ranging from “completely true” to “completely false”. Even though there is no cutoff point indicating a low or high self-efficacy level, the mean score in German cancer patients was set at 30.63 [46]. Therefore, a score lower than 29 is here considered as a low self-efficacy level. Scholz has shown good psychometric properties (Cronbach alpha of 0.85) of the GSE Scale in Dutch subjects, confirming it to be a valid instrument to examine self-efficacy [47].

##### Self-management

We will assess the survivor’s self-management level with the 27-item disease-specific Self-Management Screening (SeMaS) questionnaire [48]. It screens in which way the survivor is capable of self-management in case of chronic diseases and when confronted with difficult situations. At baseline, all 27 items will be included, distributed over the subscale education (1 item), burden of disease/late effects (1 item), control coordination (3 items), own effectiveness (2 items), social support (1 item), coping style (9 items), fear (4 items), depression (3 items) and skills such as computer skills (3 items). At T1 and T2, the education subscale is omitted. Response scales range from a 4-point Likert-type scale (“completely disagree” to “completely agree”) to a 5-point Likert scale (“no” to “very often or all the time”). Items will be scored according to the manual. In addition, a personal profile will be created based on aspects that are important for self-management. Each aspect is divided into three categories: (i) capable of (more) self-management, (ii) capable of self-management with minor barriers and (iii) major barrier(s) for (more) self-management. For the psychometric characteristic coping (problem-solving) and self-efficacy, Cronbach alpha for internal consistency was found to be acceptable (0.70) and good (0.86), respectively [48].

##### Lifestyle

To assess lifestyle changes over time, we will provide the survivors the “Leefstijlvragenlijst” questionnaire. The questionnaire is comprised of a compilation of existing validated questionnaires: the Fragerström Test for Nicotine Dependence, the short version of the International Physical Activity Questionnaire (IPAQ), a questionnaire on eating habits and the Alcohol Use Disorders Identification Tests (AUDIT) [37–40]. In addition, items on the survivor’s motivation level to improve smoking behaviours, physical activity levels, eating habits, alcohol use and weight are also included in the questionnaire [40]. To gain more insight in the physical activity level of the survivors, we will assess physical activity as a lifestyle factor separately with the SQUASH questionnaire. The validated SQUASH questionnaire includes 4 domains on commuting activities, physical activity at work or school, household activities and spare time [41]. Wendel-Vos showed the SQUASH to be a fairly reliable and reasonable questionnaire with a Spearman correlation coefficient for overall reproducibility of 0.58 in Dutch subjects (95% CI 0.36–0.74) [41].

### Analyses

#### Feasibility evaluation

To analyse qualitative data from interviews with survivors and medical professionals, the qualitative data analyses and research software Atlas.ti will be used. The REVIVER study uses grounded theory as methodological orientation to explore the survivors’ and medical professionals’ experiences with the REVIVER interventions. Therefore, an inductive approach to data analyses will be applied. Prior to analyses, the audio-recorded interviews will be fully transcribed verbatim. Subsequently, a first interpretation of the transcription will be made by thoroughly reading the transcript. Next, the transcript will be encoded by two independent coders, after which the codes will be clustered into subthemes. Any disagreements between coders will be discussed until consensus has been reached. If necessary, a third coder will be consulted to reach a consensus. Lastly, the subthemes will be grouped into major themes.

#### Potential effectiveness evaluation

All analyses will be conducted with the statistical software program SPSS (v25). Descriptive analyses will be conducted using mean with standard deviation, median with interquartile range and frequency for baseline characteristics. These variables include gender, age at intervention and treatment, socio-economic status, previous cancer diagnosis and treatment received. The validated questionnaires will be scored and analysed according to the instructions given by the authors of the questionnaires [49]. Potential covariates such as duration and amount of sessions per survivor will be presented as mean and standard deviation as well. To examine differences in the outcome measures over time, a mixed effect model with random intercept and slope will be used. In total, two nurses are assigned to deliver the interventions. The nurse who delivers the interventions may influence the survivor’s outcome. Therefore, nurse is here considered as a random effect. The outcome measures will serve as independent variables in this model with time and potential covariates as dependent variables. Tukey’s HSD test will be used as well to adjust for multiple comparisons. Due to limited power of our study, we will report data with 95% confidence intervals where needed. In addition, all data of our hypothesis testing will be treated as preliminary and with caution. Lastly, we will analyse all data according to the intention-to-treat analyses.

## Discussion

Health-related quality of life can be seriously affected in CAYA cancer survivors coping with late effects of their cancer treatments. This illustrates the necessity of high-quality and multidisciplinary cancer survivorship care to either prevent or treat late effects or to preserve the survivor’s health. We believe, inherent to the PCC principles applied at our cancer survivorship care clinic, that a survivor should no longer be seen as a patient to which care is prescribed by a doctor but as an individual taking control of their own health and health behaviours. In that line of thought, continuing life as normally as possible is rather essential for survivors. However, for some survivors, interventions are necessary to cope with late effects. Therefore, screen-to-screen eHealth interventions designed according to the survivors’ own preferences to relief them from unnecessary clinic visits seem promising. Our study is motivated by the potential value of eHealth interventions for this relatively young population. In addition, we believe that PCC, as the core principle of the REVIVER interventions, as well as the evidence-based content of all three types of interventions (CBT and/or MI), will be important contributing factors in improving the participant’s quality of life.

The decision to design interventions for CRF, self-efficacy and self-management and lifestyle is primarily based on the doctors’ and nurses’ experiences from the outpatient clinic. In addition, literature has stated the severity of CRF as a late effect of treatment, whereas maintaining or adopting a healthy lifestyle is crucial to prevent development of late effects [9–18]. On the other hand, a low self-efficacy status, due to a history of cancer, can have a negative impact on the survivor’s psychosocial domain. Likewise, when navigating through the healthcare system, low self-efficacy or self-management can be a real obstacle for the survivor. With regard to the outpatient clinic visit at the Centre of Expertise for Cancer Survivorship, survivors benefit the most of PCC when their self-efficacy level is sufficient. Therefore, we believe that targeting these specific survivorship difficulties will yield the most benefits.

The mixed methods design to evaluate the REVIVER interventions, including qualitative interviews and quantitative questionnaires, will enable us to gain a broad and in-depth insight in the feasibility and potential effectiveness of these interventions. Most importantly, we will be able to identify areas for improvement. This is paramount for further implementation of the interventions and designing a randomized controlled trial to assess (cost-)effectiveness of the REVIVER interventions on a larger scale.

However, the REVIVER study has some limitations. A first note of caution is due to the fact that we lack a control group for comparisons. Given that the interventions are implemented as care as usual at our cancer survivorship clinic, we believe it is not ethical to withhold survivors from receiving this care. Therefore, for our study, we did not opt for a control group. Instead, we will apply a pre-post design with three measurement points to gain insight in the potential effectiveness of the REVIVER interventions. Another limitation is inherent to the small nature of this feasibility study, which impedes us in providing robust data on potential effectiveness of the interventions. Notwithstanding these limitations, this study will offer us important insights into the feasibility of the REVIVER interventions.

In conclusion, given the content of the REVIVER interventions with PCC as core principle, as well as the mode of delivery, the REVIVER interventions may be a feasible and potential effective tool in delivering interventions as part of cancer survivorship care to treat or prevent late effects of cancer.

## Data Availability

The datasets used and/or analysed during the current study are available from the corresponding author on reasonable request.

## Abbreviations

AUDIT: Alcohol Use Disorders Identification Tests
BMI: Body mass index
CAYA: Childhood, adolescent and young adult
CBT: Cognitive behavioural therapy
CIS: Checklist Individual Strength
CRF: Cancer-related fatigue
eHealth: Electronic health
GSE: General Self-Efficacy
HRQOL: Health-related quality of life
IPAQ: International Physical Activity Questionnaire
MI: Motivational interviewing
PCC: Person-centred care
REVIVER: Nu*r*se-led web-based *v*ideo-coaching *i*nter*ve*ntions for su*r*vivors
SeMaS: Self-Management Screening
SQUASH: Short Questionnaire to Assess Health-enhancing physical activity
